# *Trans*-(±)-Kusunokinin Binding to AKR1B1 Inhibits Oxidative Stress and Proteins Involved in Migration in Aggressive Breast Cancer

**DOI:** 10.3390/antiox11122347

**Published:** 2022-11-27

**Authors:** Tanotnon Tanawattanasuntorn, Thidarath Rattanaburee, Tienthong Thongpanchang, Potchanapond Graidist

**Affiliations:** 1 Department of Biomedical Sciences and Biomedical Engineering, Faculty of Medicine, Prince of Songkla University, Songkhla 90110, Thailand; 2 Department of Chemistry and Center of Excellence for Innovation in Chemistry (PERCH-CIC), Faculty of Science, Mahidol University, Bangkok 10400, Thailand

**Keywords:** *trans*-(±)-kusunokinin, AKR1B1, breast cancer

## Abstract

Synthetic *trans*-(±)-kusunokinin ((±)KU), a potential anticancer substance, was revealed to have an inhibitory effect on breast cancer. According to the computational modeling prediction, AKR1B1, an oxidative stress and cancer migration protein, could be a target protein of *trans*-(−)-kusunokinin. In this study, we determined the binding of (±)KU and AKR1B1 on triple-negative breast and non-serous ovarian cancers. We found that (±)KU exhibited a cytotoxic effect that was significantly stronger than zopolrestat (ZP) and epalrestat (EP) (known AKR1B1 inhibitors) on breast and ovarian cancer cells. (±)KU inhibited aldose reductase activity that was stronger than *trans*-(−)-arctiin ((−)AR) but weaker than ZP and EP. Interestingly, (±)KU stabilized AKR1B1 on SKOV3 and Hs578T cells after being heated at 60 and 75 °C, respectively. (±)KU decreased malondialdehyde (MDA), an oxidative stress marker, on Hs578T cells in a dose-dependent manner and the suppression was stronger than EP. Furthermore, (±)KU downregulated AKR1B1 and its downstream proteins, including PKC-δ, NF-κB, AKT, Nrf2, COX2, Twist2 and N-cadherin and up-regulated E-cadherin. (±)KU showed an inhibitory effect on AKR1B1 and its downstream proteins, similar to siRNA–AKR1B1. Interestingly, the combination of siRNA–AKR1B1 with EP or (±)KU showed a greater effect on the suppression of AKR1B1, N-cadherin, E-cadherin and NF-κB than single treatments. Taken together, we concluded that (±)KU-bound AKR1B1 leads to the attenuation of cellular oxidative stress, as well as the aggressiveness of breast cancer cell migration.

## 1. Introduction

In 2020, with an expected 2.3 million new cancer patients, breast cancer has replaced lung cancer as the top cause of worldwide cancer incidence, accounting for 11.7 percent of all cancer cases [1]. Although the mortality rate from breast cancer in Europe and America is declining due to improved treatments and increased rates of early-screening diagnostics [2,3], in underdeveloped countries, there is still a low survival rate and most tumors are late stage (III/IV) at diagnosis, because patients are unable to access mammography screening tests [1]. Moreover, ovarian cancer is the most lethal gynecological cancer and is associated with breast cancer through similar multiple molecular pathways of oncogenesis, especially in hereditary breast and ovarian cancer (HBOC) syndrome. Recently, ovarian cancer has grown in incidence and its mortality rate ranked on the top-10 list of ASR and ASMR in 2020 [1].

Cancer cell migration drives metastatic aggressiveness, an important factor in the rapid transition to late-stage cancer, and results in a poor prognosis. Triple-negative breast cancer (TNBC) and non-serous ovarian carcinomas (NS) are types of cancer with aggressive migration and invasion, which generally result in a poor response and rapid progression [4,5]. Chemotherapy is a general treatment for TNBC and NS; it is most effective when a combination of drugs is used. However, multidrug resistance and strong side effects remain important problems that result in chemotherapeutic failure. Recently, targeted therapy has played a crucial role in cancer treatment due to its precision in identifying and attacking cancer cells. Targeted drugs against human epidermal growth factor receptor 2 (HER2) are widely used in HER2-positive breast and ovarian cancers. However, TNBC lacks HER2, as well as progesterone receptor (PR) and estrogen receptor (ER), leading to hormonal and HER2-targeted therapeutic failure [6]. Other tyrosine kinase receptors are considered to be potential target molecules, such as EGFR, VEGFR and Trk. In addition, a PARP inhibitor, namely, Olaparib, is usually used for treatment in HBOC patients who have BRCA1/2 mutations [7]. Unfortunately, most targeted drugs are unaffordable in economically developing nations due to their very high costs [8].

The synthetic racemic *trans*-(±)-kusunokinin ((±)KU) showed the inhibition of proliferation through significantly decreased topoisomerase II and cyclin D1 and increased p21 on breast, colon, cholangiocarcinoma and ovarian cancer cell lines [9,10]. In addition, *trans*-(−)-kusunokinin ((−)KU) inhibits tumor growth and migration with no side effects in NMU-induced mammary tumor rats [11]. (−)KU mainly binds to colony-stimulating factor 1 receptor (CSF1R) to suppress breast cancer proliferation [12], while some *trans*-(−)-kusunokinin and *trans*-(+)-isoforms could inhibit intracellular proteins, such as heat-shock protein 90 alpha (Hsp90-α) and aldo-keto reductase family 1 member B1 (AKR1B1) [13]. Interestingly, AKR1B1 was predicted to be a potential (−)KU target with a similarly stabilized orientation in the active binding pocket to CSF1R using π–π interactions. Moreover, γ-butyrolactone of (−)KU and other lignans were predicted to show the same properties as the carboxylic or hydantoin group of well-known AKR1B1 inhibitors (ARIs), namely, the ability to bind the catalytic site on AKR1B1 [14].

AKR1B1, a polyol pathway enzyme, catalyzes glucose to sorbitol. An enhancement in AKR1B1 activity induces cellular oxidative stress via the generation of lipid peroxidation and the induction of PKC/NF-κB-mediated inflammation [15]. Moreover, AKR1B1 regulates cancer migration and invasion through the conversion of prostaglandin H_2_ (PGH_2_) to prostaglandin F_2α_ (PGF_2α_) and the activation of the epithelial–mesenchymal transition (EMT) process [16]. Growing recent evidence has suggested that AKR1B1 is overexpressed in various human cancers, such as colon, liver, pancreas, lung, prostate, cervix, breast and ovarian cancer [17]. Currently, Epalrestat is the only AKR1B1 inhibitor (ARI) that has been approved by the FDA and is used for metastatic TNBC in phase II clinical trials [18]. However, its cytotoxic effect is restricted (IC_50_ value of 28.83 μg/mL on MDA-MB-231) due to its short half-life and poor hydrophilic nature [19]. Moreover, its adverse effects remain a vital problem [20]. Therefore, in order to reduce the aggressiveness of cancer, AKR1B1 is an important target for drug development.

Herein, we attempt to investigate the (±)KU targeting toward AKR1B1-mediated oxidative stress responses and the EMT process in TNBC and NS. The results from this study could provide insight into the effects of the mechanism of action of AKR1B1 inhibition on cancer and contribute data for further clinical use in the treatment of breast and ovarian cancers with aggressive migration and, thus, provide a new opportunity for chemotherapeutic agent combinations.

## 2. Materials and Methods

### 2.1. Cell Culture Condition

Human breast cancer cell lines (BT549, Hs578T and MCF7 cells) and ovarian cancer cell lines (SKOV3 and OVCAR3 cells) were purchased from ATCC (Manassas, VA, USA) while A2780 was obtained from ECACC (Salisbury, Wiltshire, UK). TNBC cell lines (BT-549 and Hs578T cells) and non-serous ovarian cancer cell lines (A2780 and SKOV3 cells) are classified as highly aggressive migration cell lines. Luminal A breast cancer cell line (MCF7 cells) and high-grade serous ovarian cancer cell line (OVCAR3 cells) are defined as the low-potential migration cell line [5,21]. BT-549, MCF-7, SKOV3, A2780 and OVCAR3 cells were maintained in RPMI-1640 medium. Hs578T cells were maintained in DMEM medium. The medium was supplemented with 10% bovine serum albumin (20% for OVCAR3), 1% L-glutamine and 1% penicillin–streptomycin (Gibco, Grand Island, USA). All cell lines were incubated in a 5% CO_2_ atmosphere, at 37 °C and 96% relative humidity.

### 2.2. Substances

*Trans*-(±)-kusunokinin ((±)KU) was synthesized as previously reported [9]. *Trans*-(−)-arctiin ((−)AR) (CAS No. 20362-31-6) was purchased from MedChemExpress (NJ, USA). Zopolrestat (ZP) (CAS No. 110703-94-1), epalrestat (EP) (CAS No. 82159-09-9), doxorubicin (DOX) (CAS No. 25316-40-9) and cisplatin (CIS) (CAS No. 15663-27-1) were obtained from Sigma-Aldrich (Saint Louis, MO, USA).

### 2.3. Cell Viability

Cell viability was performed by MTT assay as previously described [22]. BT549, Hs578T, MCF7, A2780, SKOV3 and OVCAR3 cells were seeded on a 96-well plate at a density of 3.0 × 10^4^, 2.5 × 10^4^, 2.0 × 10^4^, 1.0 × 10^4^, 2.0 × 10^4^ and 3.7 × 10^4^ cells/well, respectively, for 24 h before being treated with various concentrations of (±)KU, (−)AR, ZP, EP, DOX and CIS for 72 h. Cell viability measured the absorbance at 570 and 650 nm and calculated the half-maximal inhibitory concentration (IC_50_) value using a Varioskan™ LUX Multimode Microplate Reader (ThermoFisher Scientific, Waltham, MA, USA).

### 2.4. Aldose Reductase Activity Assay

Aldose Reductase Inhibitor Screening Kit (BioVision, CA, USA) was used to measure the aldose reductase activity toward (±)KU, (−)AR (lignan that reported as ARI), ZP (potential ARI), EP (positive control) and DMSO (solvent control). The procedure was conducted in accordance with the manufacturer’s instructions. Briefly, aldose reductase enzyme solution was added to a 96-well plate with sodium phosphate buffer (pH 6.2), 0.3 mM NADPH and tested compounds or DMSO. After 20 min incubation at 37 °C, substrate (glucose) was added and we immediately monitored a reduction in the absorbance reading at OD 340 nm in the kinetic mode for 100 min at 37 °C by the Varioskan™ LUX Multimode Microplate Reader (Thermo Fisher Scientific, MA, USA). All readings were subtracted by background control (enzyme free) and calculated using the following equation:

$$\%\;\mathrm{Relative}\;\mathrm{activity}=\frac{\left|\mathrm{Slope}\;\mathrm{of}\;\left[\mathrm{Tested}\;\mathrm{compound}\right]\right|}{\left|\mathrm{Slope}\;\mathrm{of}\;\left[\mathrm{Enzyme}\;\mathrm{control}\right]\right|}$$

### 2.5. Cellular Thermal Shift Assay (CETSA)

Hs578T and SKOV3 cells were selected due to their high expression of AKR1B1. The procedure was followed as previously described [23]. Briefly, cells with a density of 2 × 10^6^ cells/mL were treated with 5, 10 and 20 μM (±)KU or 0.002% DMSO (control) in a CO_2_ incubator at 37 °C for 1 h. Cells were harvested in 1 mL PBS containing protease inhibitor cocktail (Sigma-Aldrich, Saint Louis, MO, USA). Cell suspensions were distributed with 100 μL into 0.2 mL PCR tubes and heated at 60 or 75 °C for 3 min and cooled down at 25 °C for 3 min. The heated cell suspensions were immediately snap-frozen in liquid nitrogen and kept at −80 °C overnight. Cells were lysed by two freeze–thaw cycles using liquid nitrogen and centrifuged at 20,000× *g* for 20 min at 4 °C. Supernatants were analyzed for the AKR1B1 protein level by Western blot analysis.

### 2.6. Thiobarbituric Acid Reactive Substances (TBARS) Assay

Hs578T cells were seeded on 6-well plates at a density of 5 × 10^5^ cells/well for 24 h before being treated with various concentrations of (±)KU or EP for 24 h. After that, cells induced lipid peroxidation by 100 mM glucose for 1 h. Cells were harvested and resuspended at 1 × 10^7^ cells/mL in PBS and sonicated with 50% amplitude, pulse on/off 2 s, time 1 min, 2 cycles. The quantitation of malondialdehyde (MDA), a marker of oxidative stress, was measured using the OxiSelect™ TBARS Assay kit (Cell Biolabs, San Diego, CA, USA), in accordance with the manufacturer’s protocol. Briefly, 50 μL cell suspension was mixed with 50 μL SDS lysis solution. Samples were incubated at room temperature for 5 min and followed by adding 125 µL of thiobarbituric acid (TBA) reagent. Then, samples were incubated at 95 °C for 60 min, chilled on ice for 5 min and centrifuged at 3000× *g* for 15 min. The supernatants were transferred to a 96-well microplate and read with a spectrophotometric plate reader at 532 nm.

### 2.7. Western Blot Analysis

Hs578T cells were seeded on 24-well plates at a density of 7 × 10^4^ cells/well for 24 h. Then, cells were treated with (±)KU and EP at a concentration of 0.25 × IC_50_ and 0.50 × IC_50_ for 48 h before harvesting. The protein level of AKR1B1 and its downstream signaling cascade (PKC-δ, NF-κB, AKT, STAT3, COX2, Nrf2, Twist2, E-cadherin, N-cadherin) were detected by Western blot analysis as previously described [24]. Briefly, cells were lysed and protein extracted using RIPA buffer (Pierce, IL, USA). The amount of protein was quantified by Bradford assay (Bio-Rad, CA, USA). Fifteen micrograms of proteins was loaded into a 12% SDS-PAGE and transferred to a nitrocellulose membrane. The membrane was blocked with 5% BSA in 1 × TTBS buffer followed by incubation with primary antibodies, including anti-AKR1B1 (1:1000), β-actin (1:5000), twist2 (1:1000) (Sigma-aldrich, Saint Louis, MO, USA), N-cadherin (1:1000), NF-κB (1:500), COX2 (1:500), AKT (1:1000), PKCδ (1:1000), STAT3 (1:2000) (Cell Signaling Technology, Danvers, MA, USA), Nrf2 (1:1000) (Santa Cruz Biotechnology, Dallas, TX, USA) and E-cadherin antibodies (1:5000) (BD Transduction Laboratories, Lexington, KY, USA). Finally, bound antibodies were detected via a chemiluminescence assay kit (Thermo Scientific, Rockford, IL, USA) and visualized using a CCD camera. The band intensity was analyzed by Image J (NIH, Bethesda, MD, USA).

### 2.8. Small Interfering RNA Condition

AKR1B1 gene silencing was performed using 2 siRNA-AKR1B1 duplexes (Cat No: 4390824; targeted exons 8, 9; 5′-UCAGUUCAAAGUCAAAGACCT-3′ and Cat No: 492420; targeted exon 5; 5′-UUAACUGCAGGCUUAUACUTC-3′, Ambion, CA, USA) with DharmaFECT 4 transfection reagent (Dharmacon, CO, USA). siGENOME non-targeting control (Horizon Discovery, Waterbeach, Cambridge, UK) targets firefly luciferase mRNA and was used as a negative control. siRNA transfection was performed in accordance with the instructions. Briefly, Hs578T cells were seeded on 24-well plates at a density of 7 × 10^4^ cells/well for 24 h before transfection with 100 nM siRNA-AKR1B1 or 100 nM siGENOME non-targeting control for 24 h. Then, cells were treated with 2.79 μM (±)KU or 48.04 μM EP for 48 h. Treated cells determined the protein level of AKR1B1 and its downstream molecules using Western blot analysis.

### 2.9. Statistical Analysis

All data are represented as the mean ± standard deviation (SD). All results were determined in three independent experiments. The statistically significant differences between the two data sets were analyzed by the Student’s *t*-test, while multiple groups were analyzed by one-way analysis of variance (ANOVA) followed by Tukey’s multiple comparison test using Prism GraphPad 8.0.1 software (San Diego, CA, USA). A *p*-value of less than 0.05 was considered to indicate a statistically significant difference.

## 3. Results

### 3.1. AKR1B1 Overexpression Correlated with Aggressive Migration Cell Type (TNBC and NS Cells) and Poor Prognosis

**Figure 1:**
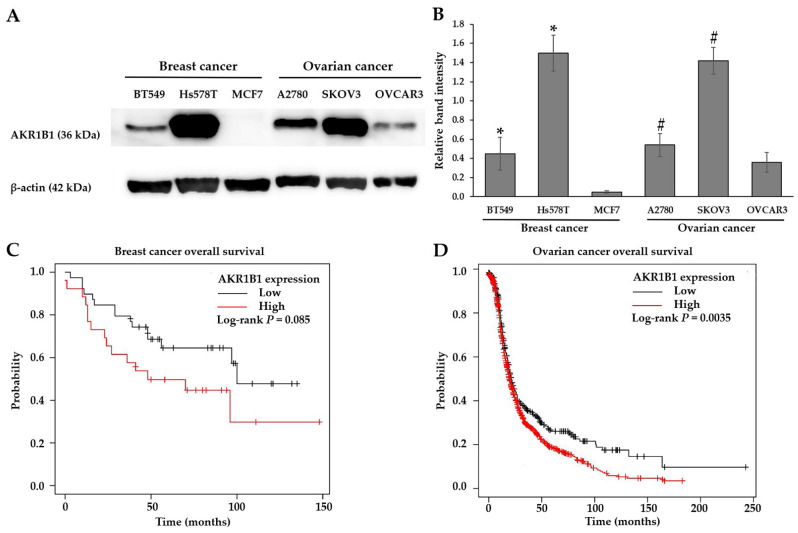
AKR1B1 overexpression correlated with the aggressive migration cell type and poor prognosis. Cells were grown for 72 h and protein was extracted using RIPA buffer. Fifteen micrograms of protein was loaded into SDS-PAGE, followed by determining the AKR1B1 level using Western blot analysis. (**A**) Protein level of AKR1B1 on the breast (BT549, Hs578T and MCF7) and ovarian (A2780, SKOV3 and OVCAR3) cancer cells. (**B**) Quantitative protein level of AKR1B1 was normalized with β-actin band intensity. Data are represented as mean ± SD with three independent experiments. Statistically significant differences were determined by the student’s *t*-test (* *p* < 0.05 versus MCF7, # *p* < 0.05 versus OVCAR3). Survival analyses of the breast (**C**) and ovarian cancer patients (**D**) were retrieved from a public database (KM-plotter).

Breast and ovarian cancer cell lines with different subtypes were detected to have AKR1B1 protein levels by Western blotting. In the breast cancer cell group, we found that TNBC cells (BT-549 and Hs578T cells) had higher AKR1B1 protein levels than luminal A subtype (MCF7 cells), and Hs578T cells showed the most distinct AKR1B1 protein. In the ovarian cancer cell group, NS cell lines (A2780 and SKOV3 cells) revealed an AKR1B1 protein higher than the high-grade serous ovarian cancer cell lines (OVCAR3 cells), and SKOV3 cells showed the highest AKR1B1 level (Figure 1A,B and Figure S1). These results indicated that the aggressive migration subtype cell lines (TNBC and NS cells) showed a high protein level of AKR1B1. Survival analysis from the KM-plotter database of breast and ovarian cancer patients revealed that the median survival rates of the high-AKR1B1 cohort were 48 and 19.3 months, respectively, while the median survival rates of the low-AKR1B1 cohort were 100 and 21 months, respectively. These results suggested that breast and ovarian cancer patients with a higher AKR1B1 expression had poorer prognostic outcomes compared to a lower AKR1B1 expression (Figure 1C,D).

### 3.2. Cytotoxic Effect of (±)KU on Breast and Ovarian Cancer Cells

**Figure 2:**
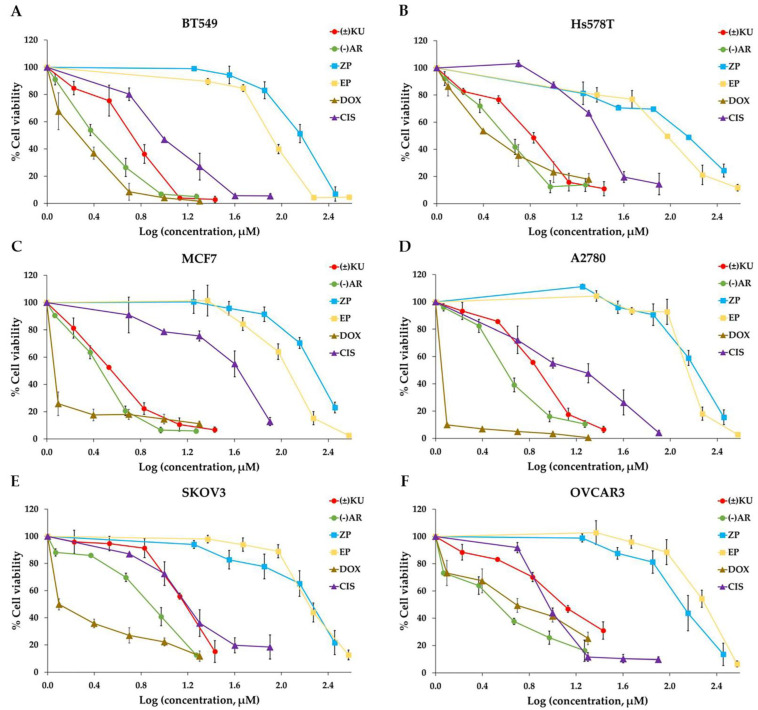
Cell viabilities of breast and ovarian cancer cells after exposure to, (±)KU, (−)AR, ZP, EP, DOX and CIS. Cells were seeded on 96-well plates for 24 h, followed by the treatment of indicated compounds for 72 h. Cell viability was determined by MTT assay. Graph representing the percentage of cell viabilities on breast cancer cells, including (**A**) BT549, (**B**) Hs578T, (**C**) MCF7 and ovarian cancer cell lines, including (**D**) A2780, (**E**) SKOV3, (**F**) OVCAR3. All data are represented as mean ± SD with three independent experiments. (±)KU, *trans*-(±)-kusunokinin; (−)AR, *trans*-(−)-arctiin; ZP, zopolrestat; EP, epalrestat; DOX, doxorubicin; CIS, cisplatin.

Cytotoxicity of (±)KU against breast and ovarian cancer cell lines with different AKR1B1 was measured by MTT assay after 72 h treatment. The IC_50_ value of (±)KU was compared with well-known ARIs (ZP and EP), lignan that was reported as ARI ((−)AR) and chemotherapeutic drugs (DOX and CIS). The results showed that the IC_50_ value of (±)KU revealed the potential cytotoxicity with IC_50_ values less than 10 μM in MCF7, Hs578T, BT549 and A2780 cells, except in SKOV3 and OVCAR3 cells. Moreover, (±)KU showed the strongest cytotoxicity on MCF7 and A2780 for breast and ovarian cancer, respectively. Interestingly, (±)KU showed a cytotoxic effect with an IC_50_ value less than well-known ARIs on both cell lines (Table 1). We observed that the dose–response curve of cell viability revealed a decreased percentage of cell viability with dose dependence in all substances. Moreover, well-known ARIs showed distinct curves from (±)KU, (−)AR, DOX and CIS on all the cell lines (Figure 2A–F).

### 3.3. Aldose Reductase Activity

**Figure 3:**
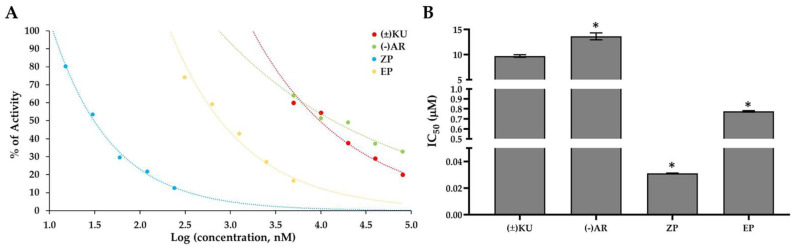
Inhibitory effect of (±)KU against aldose reductase activity. The mixtures of aldose reductase enzyme, sodium phosphate buffer, NADPH and each tested compound ((±)KU, (−)AR, ZP and EP) were incubated at 37 °C for 20 min. Then, substrate (glucose) was added and we immediately monitored the reduction in the absorption of NADPH at 340 nm kinetically for 100 min at 37 °C (**A**). Progress curves of aldose reductase enzyme activity of tested compounds at various concentrations of (**B**) IC_50_ values of the tested compounds in the inhibition of aldose reductase activity. Data representation as mean ± SD with two independent experiments. Statistically significant differences in IC_50_ values of (±)KU vs. ARIs were determined using a one-way analysis of variance followed by Tukey’s multiple comparison test (* *p* < 0.05). (±)KU, *trans*-(±)-kusunokinin; (−)AR, *trans*-(−)-arctiin; ZP, zopolrestat; EP, epalrestat.

In order to evaluate an aldose reductase inhibitor effect of (±)KU, we measured the aldose reductase activity by monitoring the reduction in NADPH. To ensure that DMSO, the solvent of (±)KU, had no effect on the reaction, the progress curves of the reduction in NADPH in the reaction with or without DMSO (solvent control and enzyme control, respectively) were plotted to compare the aldose reductase activity and revealed that the slopes of both curves were not significantly different (Figure S2). Moreover, enzyme free (background control) was used to subtract the slope and EP was used as a positive control. Aldose reductase activity was plotted in the progress logarithmic curves of the percentage of activity at the various concentrations of substances ((±)KU, (−)AR, ZP and EP) in nanomolar (nM). We observed that all the substances inhibited aldose reductase activity in a dose-dependent manner (Figure 3A). (±)KU showed potential inhibition of the aldose reductase activity with an IC_50_ value of 9.72 ± 0.18 μM and showed better than the reported ARI lignan (−)AR (IC_50_ value of 13.65 ± 49 μM). Meanwhile, ZP and EP, the well-known AKR1B1 inhibitors, specifically designed for targeting aldose reductase, showed an IC_50_ value of 31.03 ± 0.14 nM and 0.77 ± 0.01 μM, respectively (Figure 3B).

### 3.4. Cellular Thermal Shift Assay (CETSA)

**Figure 4:**
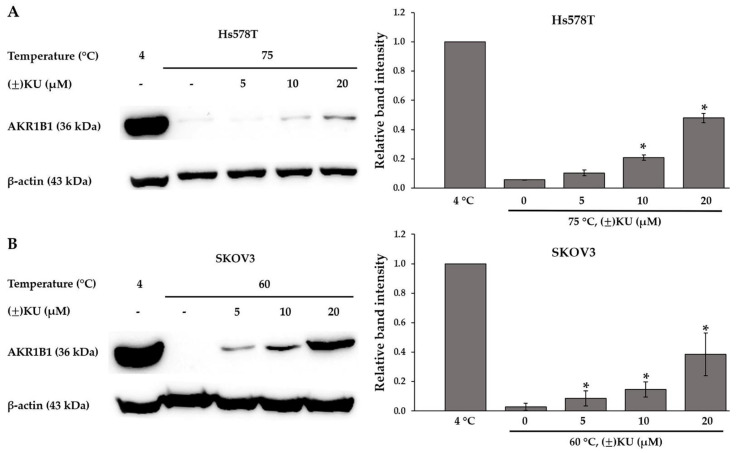
CETSA-based determination of target engagement between (±)KU and AKR1B1. (**A**) Hs578T and (**B**) SKOV3 cells were treated with various concentrations of (±)KU (5, 10 and 20 μM) at 37 °C for 1 h. Then, cells were harvested and heated at 75 and 60 °C, respectively, followed by determination using Western blot analysis. The quantitative protein level of AKR1B1 was normalized with β-actin band intensity. Data are represented as mean ± SD with three independent experiments. Statistically significant differences between treated cells and non-treated cells were determined by the student’s *t*-test (* *p* < 0.05). (±)KU, *trans*-(±)-kusunokinin.

From in silico analysis, it was predicated that (±)KU could potentially bind to AKR1B1. However, there is no in vitro evidence to prove that AKR1B1 is a direct target of (±)KU. To determine whether (±)KU can directly bind to AKR1B1, we performed the CETSA, a label-free technique to verify the target of compounds. CETSA relies on the principle that compound binding increases the thermal stabilization of the target protein. The results showed that when Hs578T and SKOV3 cell lysates were heated at 75 and 60 °C, respectively, the AKR1B1 protein was almost eliminated. While cell lysates were treated with (±)KU, AKR1B1 proteins were stabilized after heating. Cell lysates underwent no treatment of (±)KU. We observed a noticeable difference at 75 and 60 °C on Hs578T and SKOV3 cells, respectively (Figure S3). Then, we performed further analysis using these temperatures with various concentrations of (±)KU. The results showed that (±)KU enhances the stability of AKR1B1 protein levels in a dose-dependent manner on both cell lines and showed a significantly stabilized AKR1B1 protein at 10 and 20 μM of (±)KU concentration for Hs578T cell lines (Figure 4A and Figure S4), while the SKOV3 cell line showed a significantly stabilized AKR1B1 protein at 5, 10 and 20 μM of (±)KU concentration (Figure 4B).

### 3.5. (±)KU Inhibited Lipid Peroxidation in High-Glucose Condition

**Figure 5:**
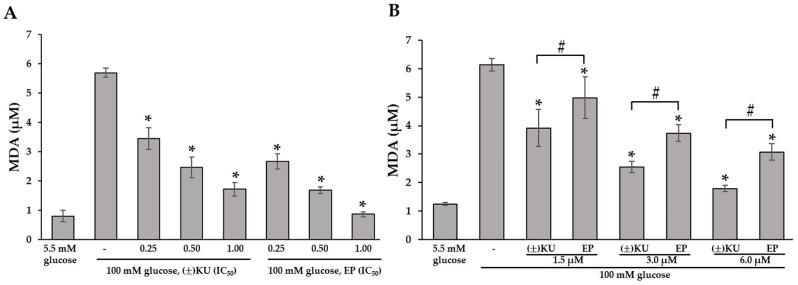
(±)KU causes the inhibition of lipid peroxidation on Hs578T in high-glucose conditions. Hs578T cells were treated with (±)KU or EP at the indicated concentration for 24 h before being replaced with RPMI medium with or without 100 mM glucose for 1 h. Cells were homogenized and MDA level measured using TBARS assay. (**A**) Quantitative MDA level on Hs578T-pretreated cells with (±)KU and EP at 0.25×, 0.50× and 1.00× IC_50_ values of cytotoxicity. (**B**) Quantitative MDA levels on Hs578T-pretreated cells with (±)KU and EP at the same concentration (1.5, 3.0, 6.0 μM). All graphs show mean ± SD with four independent experiments. Statistically significant differences for pretreated cells with various concentrations of (±)KU and EP vs. non-pretreated cells were determined using a one-way analysis of variance followed by Tukey’s multiple comparison test (* *p* < 0.05 versus only glucose treatment, # *p* < 0.05 is considered of (±)KU to compare to EP treatment). (±)KU, *trans*-(±)-kusunokinin; EP, epalrestat.

In order to evaluate the inhibitory effect of (±)KU on high-glucose-induced AKR1B1-mediated oxidative stress response, Hs578T cell lines detected lipid peroxidation using TBARS assay. Hs578T cells were selected to perform in consequence of the more potential cytotoxic effect than SKOV3 cells. Hs578T cells were incubated with (±)KU and EP at 0.25×, 0.50× and 1.00× IC_50_ (1.40, 2.79, 5.58 μM for (±)KU and 24.02, 48.04, 96.08 μM for EP, respectively) for 24 h. Then, oxidative stress was induced with a high glucose concentration at 100 mM for 1 h. After that, cells were measured for the quantitation of malondialdehyde (MDA), which form as MDA–TBA adduction. The results revealed cells that were induced with a high glucose concentration and markedly increased MDA levels compared with those not treated with high glucose. Interestingly, cells that were pretreated with (±)KU and EP showed significantly decreased MDA levels in a dose-dependent manner (Figure 5A). However, (±)KU showed decreased MDA levels, which were lower than EP when compared to the concentration of IC_50_ values of cytotoxicity on Hs578T cells, which provide different concentrations. We also further detected the MDA levels when we pretreated the cells with (±)KU and EP at the same concentration (1.5, 6.0 and 3.0 μM). We found that (±)KU showed significantly decreased MDA levels, which were better than EP (Figure 5B).

### 3.6. (±)KU Suppressed AKR1B1 Level in a Dose-Dependent Manner

**Figure 6:**
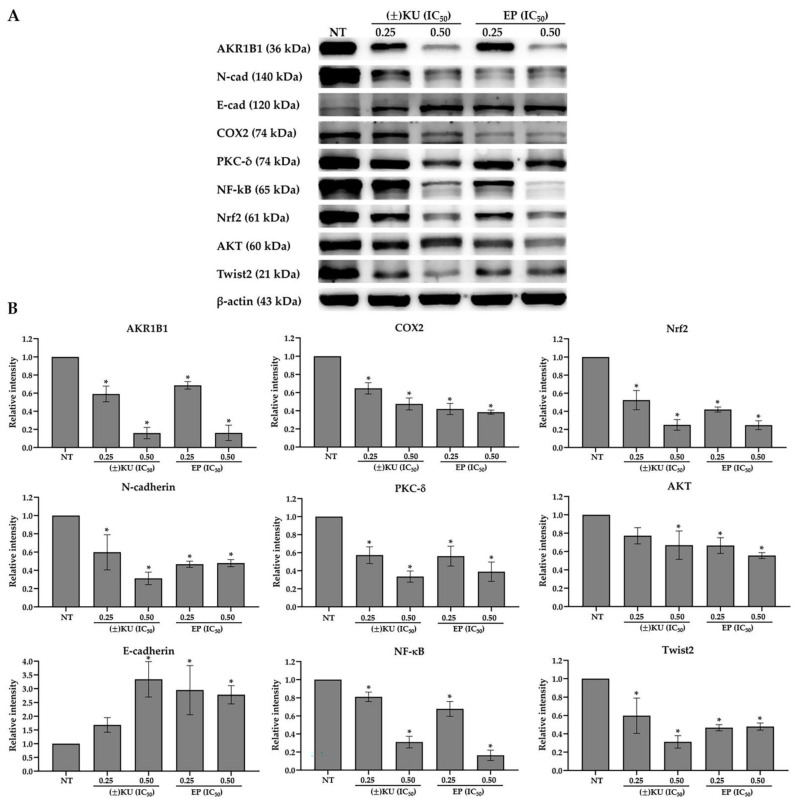
Effect of (±)KU on AKR1B1 and its downstream signaling molecules. Hs578T cells were treated with (±)KU and EP at a concentration of 0.25× and 0.50× IC_50_ (1.40, 2.79 μM for (±)KU and 24.02, 48.04 μM for EP, respectively) for 48 h. (**A**) The protein level of AKR1B1 and its downstream molecules (PKCδ, NF-κB, AKT, Nrf2, COX2, Twist2, E-cadherin and N-cadherin) were determined by Western blot analysis. (**B**) Quantitative protein level of AKR1B1 and its downstream molecules were normalized with β-actin band intensity. Data are represented as mean ± SD with three independent experiments. Statistically significant differences between treated cells and non-treated cells were determined by a one-way analysis of variance, followed by Tukey’s multiple comparison test (* *p* < 0.05). NT, non-treated cells; (±)KU, *trans*-(±)-kusunokinin; EP, epalrestat.

In order to determine the potential of (±)KU on the inhibition of AKR1B1 compared to the AKR1B1 inhibitor (EP), Hs578T cells were treated with (±)KU or EP at a concentration of 0.25× and 0.50× IC_50_ (1.40, 2.79 μM for (±)KU and 24.02, 48.04 μM for EP, respectively) for 48 h. We detected the protein level of AKR1B1 and its downstream signaling molecules PKCδ, NF-κB, AKT, Nrf2, COX2, Twist2, E-cadherin and N-cadherin. The results showed that (±)KU markedly suppressed AKR1B1 levels in a dose-dependent manner, similar to EP. Moreover, (±)KU at 0.50× IC_50_ significantly down-regulated on signaling molecules, including PKCδ, NF-κB, AKT, Nrf2, COX2, Twist2 and N-cadherin, and up-regulated on E-cadherin (Figure 6 and Figure S5). Interestingly, (±)KU showed potential for a reduction in AKR1B1 levels and its downstream signaling molecules, the same as EP inhibition at a lower concentration. These results suggest that (±)KU suppressed AKR1B1 levels, leading to a reduction in signaling molecules.

### 3.7. Down-Regulation of AKR1B1 by (±)KU Leads to Alteration in Signaling Molecules of Oxidative Stress and EMT Markers

**Figure 7:**
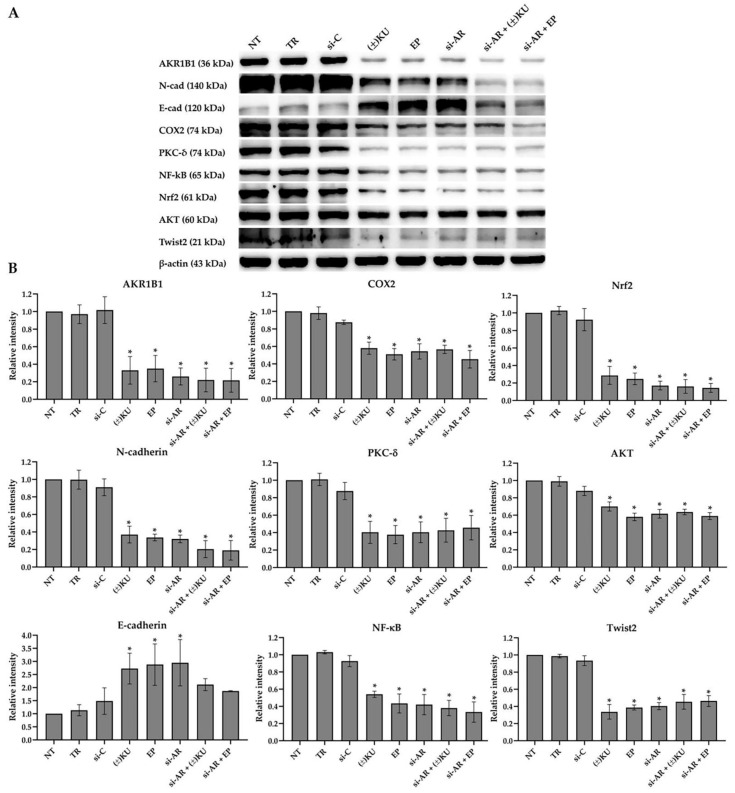
Effect of (±)KU, EP, siRNA-AKR1B1 and combination of compounds with siRNA-AKR1B1 on AKR1B1 and its downstream signaling molecules. Hs578T cells were treated with 2.79 μM (±)KU or 48.04 μM EP or 100 nM siRNA-AKR1B1 or a combination of 100 nM siRNA-AKR1B1 with each compound for 72 h. (**A**) The protein level of AKR1B1 and its downstream molecules (PKCδ, NF-κB, AKT, Nrf2, COX2, Twist2, E-cadherin and N-cadherin) were determined by Western blot analysis. (**B**) Quantitative protein level of AKR1B1 and its downstream molecules were normalized with β-actin band intensity. Data are represented as mean ± SD with three independent experiments. Statistically significant differences between treated cells and non-treated cells were determined by a one-way analysis of variance followed by Tukey’s multiple comparison test (* *p* < 0.05). NT, non-treated cells; TR, DharmaFECT 4 transfection reagent; si-C, siGENOME non-targeting control; (±)KU, *trans*-(±)-kusunokinin; EP, epalrestat; si-AR, siRNA-AKR1B1.

To clarify the mechanism of action of (±)KU on oxidative stress response and epithelial–mesenchymal transition process through the inhibition of AKR1B1, Hs578T cells were treated with 2.79 μM (±)KU or 48.04 μM EP or 100 nM siRNA-AKR1B1 or a combination of 100 nM siRNA-AKR1B1 with each compound for 72 h. In this study, untreated cells, dharmafect-transfection reagent and siRNA-luciferase treatments were considered as the negative control, while EP and siRNA-AKR1B1 treatments served as positive controls, which determined the AKR1B1 level and its downstream signaling molecules of oxidative stress and EMT markers, including PKCδ, NF-κB, AKT, Nrf2, COX2, Twist2, E-cadherin and N-cadherin. The results showed that (±)KU dramatically down-regulates AKR1B1 levels as potential EP and siRNA-AKR1B1 treatments. Interestingly, a combination of (±)KU or EP with siRNA-AKR1B1 showed a stronger suppressive effect on AKR1B1, NF-κB, Nrf2 and N-cadherin than single treatments. Moreover, E-cadherin levels were up-regulated when treated with (±)KU, EP and siRNA-AKR1B1 treatments, but decreased in the combination treatments (Figure 7 and Figure S6). These results suggested that (±)KU suppressed AKR1B1 and altered downstream signaling molecules with a similar action to EP.

## 4. Discussion

Previously, we reported that kusunokinin had a cytotoxic effect on cancer cell lines, for instance, ovarian cancer (A2780, A2780cis, SKOV3 and OVCAR3), colon cancer (HT-29), cholangiocarcinoma (KKU-M213 and KKU-K100) and several breast cancer subtypes, including luminal A (MCF7), basal-A (MDA-MB-468) and basal-B (MDA-MB-231) [9,10]. The natural (−)KU showed significantly increased apoptosis on MCF7 and MDA-MB-468 cells after treatment for 24 h. (−)KU decreased anti-apoptotic protein (bcl2) and increased apoptotic protein (bax, cytochrome c, caspase-7 cleaved and caspase-8 cleaved) on MCF-7 cells at 72 h [25]. Interestingly, the synthetic racemic (±)KU also greatly induced apoptosis on MCF7 cells in a time-dependent manner. This synthetic compound significantly enhanced multi-caspase activity (caspase-1, -3, -4, -5, -6, -7, -8 and -9) on MCF7 cells in a time- and dose-dependent manner [9]. In addition, (±)KU significantly increased the number of early/late/total apoptotic cells, multi-caspase activity and apoptotic proteins (bax and PUMA) in ovarian cancer cells (A2780 and A2780cis) at 72 h [10]. All these previous studies confirm that (±)KU had a cytotoxic effect through the induction of apoptosis.

In this study, (±)KU had IC_50_ values on ovarian cancer (A2780, SKOV3 and OVCAR3) that were similar to CIS (chemotherapeutic drug for ovarian cancer treatment) and our previous report [10]. (±)KU showed strong cytotoxicity against breast cancer cell lines on both minimally potential migration (MCF7) and aggressive migration (BT549 and Hs578T). In addition, the results revealed that (±)KU showed a similar IC_50_ value on the TNBC subtype (6.16 ± 0.32, 5.57 ± 0.23 μM on BT549 and Hs578T, respectively), which is in concordance with a previous report of the other TNBC cells (5.90 ± 0.44, 7.57 ± 0.92 μM on MDA-MB-468 and MDA-MB-231, respectively) [9]. Although (±)KU had the greatest effect on MCF7, this cell expressed the AKR1B1 at a very low level. It may result from the action of (±)KU on other target proteins. We reported that (±)KU suppressed MCF7 cell proliferation through its binding with CSF1R. Moreover, using a computational simulation, we also found that (±)KU bound MMP-12, HSP90-α, CyclinB1 and MEK1 [12,13]. Another reason for the effect of (±)KU on MCF7 is the characterization of the cells. MCF7 is the luminal A subtype; meanwhile, BT549 and Hs578T are triple-negative breast cancer subtypes. Therefore, MCF7 is weaker than BT549 and Hs578T cells [26]. In our study, we showed that DOX (a breast-cancer-specific chemotherapeutic drug) inhibited MCF7 cells with a lower IC_50_ value when compared with BT549 and Hs578T. (±)KU also showed that the inhibitory effect of all these breast cancer cells was the same as DOX. However, ZP and EP (AKR1B1 inhibitors) showed a weak cytotoxic effect on all ovarian and breast cancer cell lines, especially on MCF7 and OVCAR3 cells, which a lack of AKR1B1. Moreover, our study showed that the IC_50_ values of ZP and EP on TNBC cell lines (BT549 and Hs578T) were almost equal to another report on the cytotoxic effect of EP on MDA-MB-231. The weak cytotoxic effect of EP is a result of slightly hydrophilic properties and a short half-life, leading to its unsuitability for use as an anticancer drug [19].

(±)KU inhibits the proliferation of breast cancer cells through the suppression of CSF1R and its downstream signaling molecules, including AKT, cyclinD1 and CDK1. (−)KU was predicted to bind to the juxtamembrane region and form π–π stacking at the binding site of CSF1R [12]. Moreover, it also binds to the ATP binding domain of HER2 but revealed low binding affinity and a different mode of action from HER2 inhibitor (neratinib) [27]. Recently, (−)KU was predicted as an inhibitor of AKR1B1, the new target that plays an important role in cellular oxidative stress and the EMT process on cancer cells. (−)KU stabilizes orientation at the binding pocket of AKR1B1 using π–π stacking, which has a similar interaction with CSF1R. Interestingly, γ-butyrolactone of (−)KU showed the same binding mode of action to the carboxylic or hydantoin group of well-known ARIs, which form a hydrogen bond to the His110 catalytic site and π–π interaction with Trp111. We concluded that (−)KU could be a potential AKR1B1 inhibitor [14]. However, there is no in vitro evidence to prove these computational simulation results. Therefore, in this study, (±)KU was investigated for its direct target binding on AKR1B1 using the cellular thermal shift assay (CETSA). This technique is a label-free protein target identification strategy for active compounds that evaluate the engagement of drug and target proteins in the cells [28]. Previously, CETSA was performed in order to investigate molecular targets related to the antiosteoporosis effects of 9′-O-di-(E)-feruloyl-meso-5, 5′-dimethoxysecoisolariciresinol, a lignan isolated from *Litsea cubeba*, which revealed potential binding activities to cathepsin K and MEK1 [29]. In the present study, our results showed a significantly stabilized AKR1B1 level at concentrations of 10 and 20 μM on both Hs578T and SKOV3 cells. These results demonstrate the potential binding of (±)KU toward AKR1B1 with dose dependence as a consequence of the protection in AKR1B1 degradation from heating.

We further investigated the inhibitory effect of (±)KU on AKR1B1 enzyme activity. We found that (±)KU potentially inhibits the conversion of glucose to sorbitol by AKR1B1 enzyme activity in a dose-dependent manner and showed a stronger effect than (−)AR, a lignan compound that is reported to be an AKR1B1 inhibitor [30], and revealed a structure similar to (±)KU. Moreover, lignans extracted from several plants also revealed their potential inhibitory effect on AKR1B1 activity, including lignans extracted from *Eucommia ulmoides* [31], *Fructus arctii* [32] and *Viburnum cylindricum* [33]. These findings support the possibility that (±)KU and lignan compounds with chemical structures similar to (±)KU could be potential AKR1B1 inhibitors.

**Figure 8:**
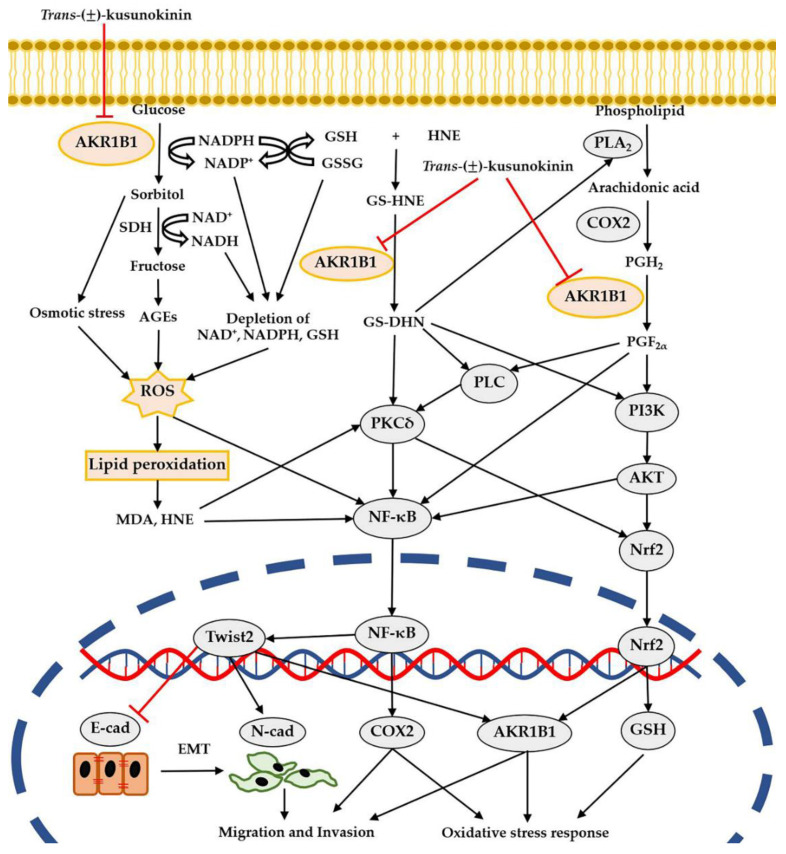
Schematic illustration of the proposed action of *trans*-(±)-kusunokinin on aggressive breast cancer.

AKR1B1 plays an important role in cellular oxidative stress through the polyol pathway that catalyzes glucose to sorbitol [34]. Generally, the metabolism of glucose by AKR1B1 through this pathway shows poor activity. However, in hyperglycemic conditions, AKR1B1 activity is stimulated, leading to an increase in the generation of reactive oxygen species (ROS) from several events; for example, the depletion of NADPH, GSH and NAD^+^ occurs from the sorbitol generation by utilizing NADPH, the conversion of oxidized glutathione to reduce glutathione (GSH) by consuming NADPH and the conversion of sorbitol to fructose by SDH through an NAD^+^-dependent reaction, respectively [15]. The accumulation of sorbitol itself leads to oxidation stress and even the conversion of sorbitol to fructose, leading to advanced glycation end products (AGEs), which latterly enhance ROS generation (Figure 8). Consequently, ROS caused oxidative damage to the membrane lipid, leading to the generation of lipid peroxidation products, such as 4-hydroxy-trans-2-nonenal (HNE) and malondialdehyde (MDA), which are stable mediators and reflect oxidative stress [35]. Previously, the protective effect of fisetin on high-glucose-induced oxidative damage in HT22 cells by decreased MDA level was reported [36]. Moreover, in vivo studies of STZ-induced diabetic rats were shown to enhance the MDA level and later the treatment of AKR1B1 inhibitors, which ameliorated oxidative stress in diabetic complications by decreasing MDA levels [37,38]. Consistent with these findings, we found that Hs578T cells that were cultivated in the high-glucose condition (100 mM) showed a significant increase in MDA level when compared to cultivation in the normal-glucose condition (5.5 mM). Interestingly, (±)KU exposure caused a significant reduction in MDA level and showed more effectiveness than EP. Since AKR1B1 plays an essential role in oxidative stress and MDA was selected as the lipid peroxidation marker, we concluded that the inhibition of AKR1B1 by (±)KU caused the reduction in oxidative stress. However, the action of (±)KU as an antioxidant should be studied further.

AKR1B1 is greatly associated with the epithelial–mesenchymal transition (EMT) process through the polyol pathway. In cancer studies, AKR1B1 strongly correlates with aggressive and invasive cancer. Schwab and colleagues reported that AKR1B1 knockdown inhibited EMT, proliferation and cancer stem cell markers on A549 non-small-cell lung cancer cells. Moreover, high-glucose-induced AKR1B1 activation enhanced migration and EMT marker alteration (E-cadherin, vimentin and ZEB1) through TGFβ stimulation [39]. In diabetic studies, the AKR1B1-induced EMT process is mediated by oxidative stress in the cataract lens of hyperglycemic patients. As a result, diabetic cataract lenses showed a significantly higher expression of AKR1B1, RAGE, 3NT, N-cadherin and MMP9 than non-diabetic cataract lenses [40]. Furthermore, AKR1B1 implicated EMT through the metabolism of lipid peroxidation-derived aldehydes. AKR1B1 converts the glutathionyl-4-hydroxynonenal (GS-HNE) to glutathionyl-1,4-dihydroxynonene (GS-DHN), which activates phospholipase C (PLC) and protein kinase C (PKC) and relays the NF-κB signaling to regulate migration and invasion [34] (Figure 8). Previous studies have demonstrated that AKR1B1 inhibition by sorbinil and zopolrestat hinders EGF/FGF-induced growth, migration and invasion through GS-DHN oxidative stress mediator-induced NF-κB activation on HT29 and KM20 colon cancer cells [41]. AKR1B1 also regulates EMT via prostaglandin biosynthesis. Generally, the phospholipid is catalyzed to arachidonic acid by phospholipase A_2_ (PLA_2_), which is feasibly activated by GS-DHN. Then, arachidonic acid is turned into prostaglandin H_2_ (PGH_2_) by cyclooxygenase 2 (COX2), which is the key downstream molecule of NF-κB signaling [34]. AKR1B1 converts PGH_2_ to prostaglandin F_2α_ (PGF_2α_), which activates PI3K/AKT signaling and possibly relays the activation of nuclear factor-erythroid-2-related factor 2 (Nrf2), which regulates AKR1B1 expression [42]. PGF_2α_ directly relays NF-κB signaling and indirectly activates NF-κB through the cascade of PLC/PKC signaling [43]. NF-κB plays an important role in the expression of Twist2, which regulates EMT markers (E-cadherin and N-cadherin) and directly transcribes AKR1B1 [16]. These studies support our current experiments. The mechanism of action of (±)KU on Hs578T cells was evaluated for its inhibitory effect on AKR1B1 and its downstream molecules (PKCδ, NF-κB, AKT, Nrf2, COX2 and Twist2), including the alteration in EMT markers (E-cadherin and N-cadherin). We found that (±)KU significantly suppressed AKR1B1 and its downstream molecules and altered EMT marker levels (up-regulation of E-cadherin and down-regulation of N-cadherin) in a dose-dependent manner. Our results show concordance with previous studies: gedunin, a limonoid compound, revealed the inhibition of AKR1B1 and its downstream (AKT, ERK and NF- κB), ROS generation and hypoxia-induced cell migration on SCC131 oral cancer cells [44]. Moreover, fidarestat, an AKR1B1 inhibitor, decreased COX2 and NF-κB on HT29 colon cancer cells and inhibited PKC, AKT and COX2 in azoxymethane-induced colonic premalignant mice [45]. We further investigated the effect of (±)KU on Hs578T cells by comparing it to AKR1B1 inhibitor (EP), siRNA-AKR1B1 and a combination of regimens. The results showed that the silencing of AKR1B1 using siRNA-AKR1B1 was not clearly suppressed due to the abundance of AKR1B1 protein on Hs578T cells (Figure 1A). We treated the cells with the maximum concentration of siRNA-AKR1B1 (100 nM), in accordance with the instructions. We would like to note that using a high concentration of siRNA could cause an off-target effect and toxicity on the cells [46]. However, we noticed that using 100 nM siRNA-AKR1B1 treatment can reduce the protein level of AKR1B1 by 74.51% compared to siRNA-control. Moreover, the protein level of the downstream molecules also showed clear alterations after the silencing of AKR1B1. We found that (±)KU inhibited AKR1B1 and its downstream molecule and altered EMT marker levels with a similar effect to EP and siRNA-AKR1B1. Moreover, the combination regimens of siRNA-AKR1B1 with (±)KU or EP showed more suppressive AKR1B1 and N-cadherin than siRNA-AKR1B1 treatment alone. Interestingly, we noticed that the single treatment of (±)KU or EP dramatically up-regulated E-cadherin levels but the combination of regimens decreased E-cadherin level, with no significant difference to the control groups. Similarly, the treatment of pexidartinib (CSF1R inhibitor) in combination with siRNA-CSF1R on MCF7 cells brought CSF1R and AKT levels back to the control baseline [12]. These results suggested that (±)KU inhibited AKR1B1 with a similar action to EP and showed a potential effect similar to siRNA-AKR1B1 treatment. Likewise, it has been reported that, for the inhibition of AKR1B1 on TNBC cancer cells (MDA-MB231 and SUM159), EP treatment and knockdown of AKR1B1 lead to an alteration in the level of EMT markers (up-regulation of E-cadherin and down-regulation of vimentin) and decreased cancer cell migration and invasion. In addition, the AKR1B1 overexpression by stable vector transfection on luminal breast cancer cells (T47D and MCF7) showed a contrary effect [16].

## 5. Conclusions

(±)KU had a potential anticancer effect on breast cancer cells and showed potential for inhibiting AKR1B1 enzyme activity. (±)KU bound to AKR1B1, leading to the stabilization of AKR1B1 protein at high temperatures. The inhibitory effect of (±)KU against AKR1B1 resulted in the protection of glucose-induced cellular oxidation, the suppression of signaling molecules and an alteration in the EMT markers on Hs578T breast cancer cells.

## Figures and Tables

**Table 1:** IC_50_ values of (±)KU, ARIs and chemotherapeutic drugs against breast and ovarian cancer cells.

Cell Lines	IC_50_ Values of the Compounds (μM)					
	(±)KU	(−)AR	ZP	EP	DOX	CIS
Breast cancer						
BT549	6.16 ± 0.32	2.52 ± 0.17	119.81 ± 1.66	82.33 ± 0.56	1.84 ± 0.16	9.69 ± 0.10
Hs578T	5.57 ± 0.23	3.41 ± 0.05	116.34 ± 2.70	96.08 ± 2.48	2.81 ± 0.16	23.39 ± 0.39
MCF7	3.55 ± 0.01	2.90 ± 0.17	191.13 ± 3.88	111.93 ± 0.24	0.69 ± 0.04	40.22 ± 0.53
Ovarian cancer						
A2780	7.35 ± 0.13	4.11 ± 0.12	156.45 ± 5.16	184.83 ± 1.68	0.14 ± 0.05	5.06 ± 0.17
SKOV3	14.34 ± 0.18	7.24 ± 0.09	121.37 ± 1.73	180.77 ± 1.29	1.26 ± 0.02	12.31 ± 0.71
OVCAR3	12.33 ± 0.23	3.42 ± 0.30	183.90 ± 4.16	191.48 ± 1.24	3.93 ± 0.02	9.84 ± 0.14

IC_50_ values showed mean ± SD from three independent experiments. (±)KU, *trans*-(±)-kusunokinin; (−)AR, *trans*-(−)-arctiin; ZP, zopolrestat; EP, epalrestat; DOX, doxorubicin; CIS, cisplatin.

## Data Availability

Data is contained within the article and supplementary material.

## Supplementary Materials

The following supporting information can be downloaded at: https://www.mdpi.com/article/10.3390/antiox11122347/s1, Figure S1: AKR1B1 protein levels on breast and ovarian cancer cells. Protein levels were determined using Western blot analysis. Images representing the results in Figure 1A and calculations for the relative intensity in Figure 1B; Figure S2: Progress curves of the aldose reductase reaction control. Aldose reductase enzyme control, enzyme-free (background control), solvent control and positive control (5 μM EP) of the reaction monitored the reduction in NADPH at OD 340 nm in kinetic mode for 100 min; Figure S3: CETSA-based determination of target engagement of (±)KU toward AKR1B1 on (A) Hs578T cells and (B) SKOV3 cells at various temperatures. Protein levels were determined using Western blot analysis; Figure S4: CETSA-based determination of target engagement of (±)KU toward AKR1B1 on (A) Hs578T cells and (B) SKOV3 cells at various concentrations of (±)KU. Protein levels were determined using Western blot analysis. (A) and (B) represent the results calculated for the relative intensity in Figure 4A and Figure 4B, respectively; Figure S5: Suppression of AKR1B1 and alteration in signaling molecules by (±)KU and EP at a concentration of 0.25× and 0.50× IC_50_ values on Hs578T cells. Protein levels were determined using Western blot analysis. Images representing the results in Figure 6A and calculations for the relative intensity in Figure 6B; Figure S6: Suppression of AKR1B1 and alteration in signaling molecules by (±)KU, EP, siRNA-AKR1B1 and the combination of siRNA-AKR1B1 with (±)KU or EP treatments on Hs578T cells. Protein levels were determined using Western blot analysis. Images representing the results in Figure 7A and calculations for the relative intensity in Figure 7B.
