# Supplementary material for: Trans-(±)-Kusunokinin Binding to AKR1B1 Inhibits Oxidative Stress and Proteins Involved in Migration in Aggressive Breast Cancer
Source: Antioxidants (Basel). 2022 Nov 27;11(12):2347. doi: 10.3390/antiox11122347 (PMC9774946; doi:10.3390/antiox11122347)
Supplement: Supplementary file 1 [file antioxidants-11-02347-s001.zip › antioxidants-1940356-supplementary.pdf]

antioxidants

MDPI

Supplementary Material

Trans-(±)-kusunokinin Binding to AKR1B1 Inhibits Oxidative Stress and Proteins Involved in Migration in Aggressive Breast Cancer

Tanotnon Tanawattanasuntorn [1], Thidarath Rattanaburee [1], Tienthong Thongpanchang [2], and Potchanapond Graidist [1,*]

[1] Department of Biomedical Sciences and Biomedical Engineering, Faculty of Medicine, Prince of Songkla University, Songkhla 90110, Thailand
[2] Department of Chemistry and Center of Excellence for Innovation in Chemistry (PERCH-CIC), Faculty of Science, Mahidol University, Bangkok 10400, Thailand
* Correspondence: gpotchan@medicine.psu.ac.th; Tel.: +66-74-45-1184

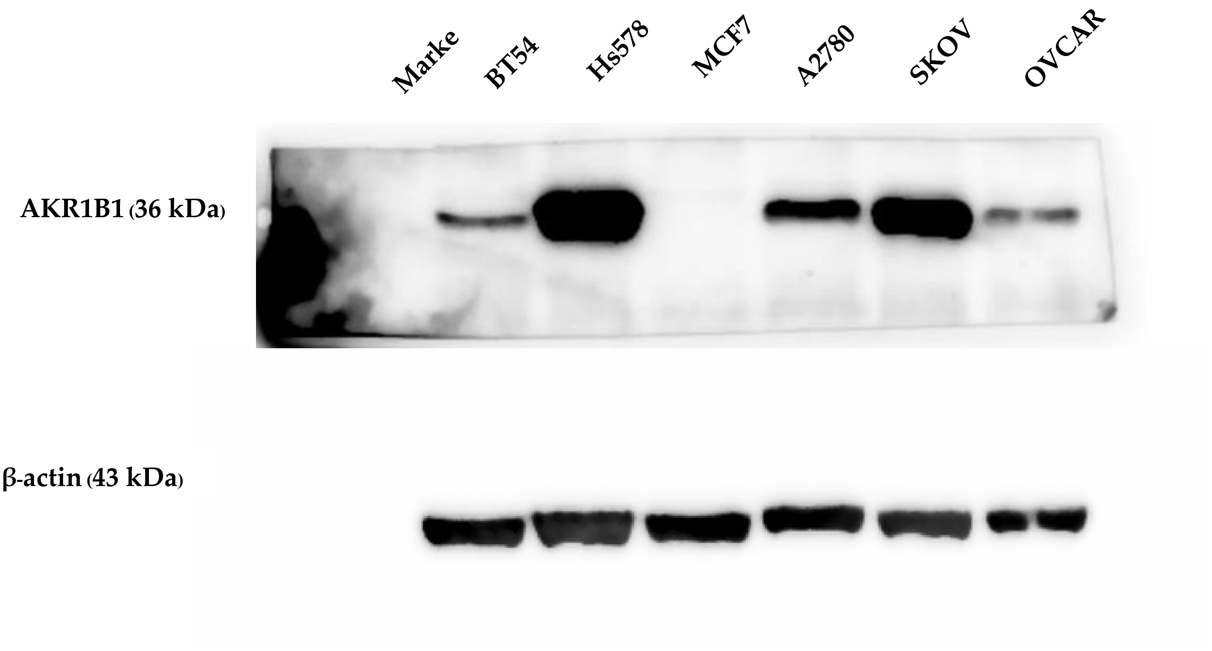

Figure S1. AKR1B1 protein level on breast and ovarian cancer cells. Protein level was determined using Western blot analysis. These images are represented in the results in Figure 1A and the calculation of the relative intensity is represented in Figure 1B.

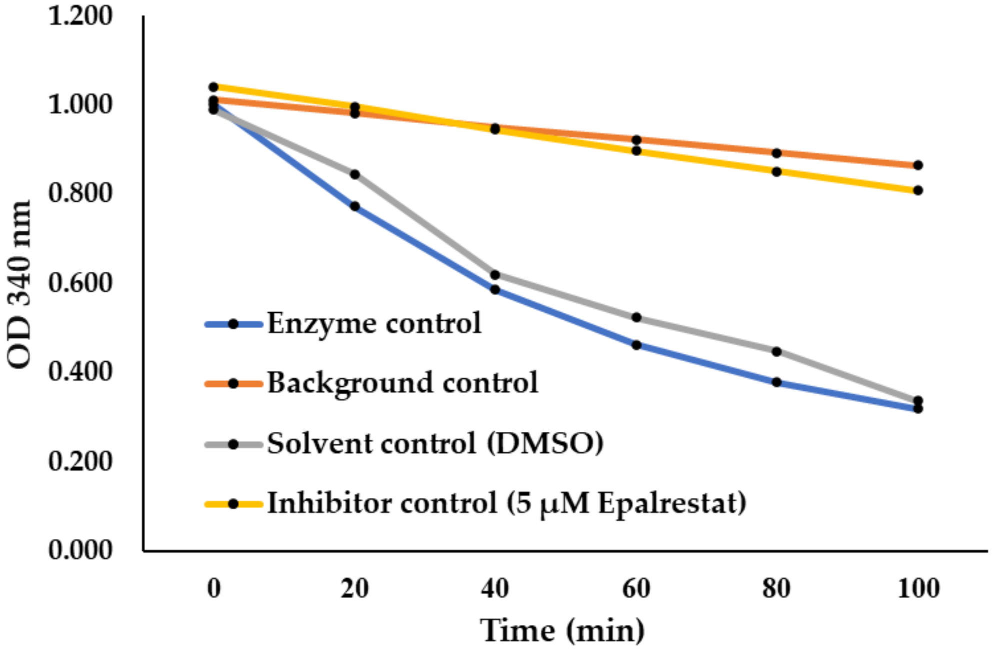

Figure S2. Progression curves of the aldose reductase reaction control. The reduction in NADPH in aldose reductase enzyme control, enzyme-free (background control), solvent control and positive control (5 μM epalrestat) were monitored at OD 340 nm in kinetic mode for 100 min.

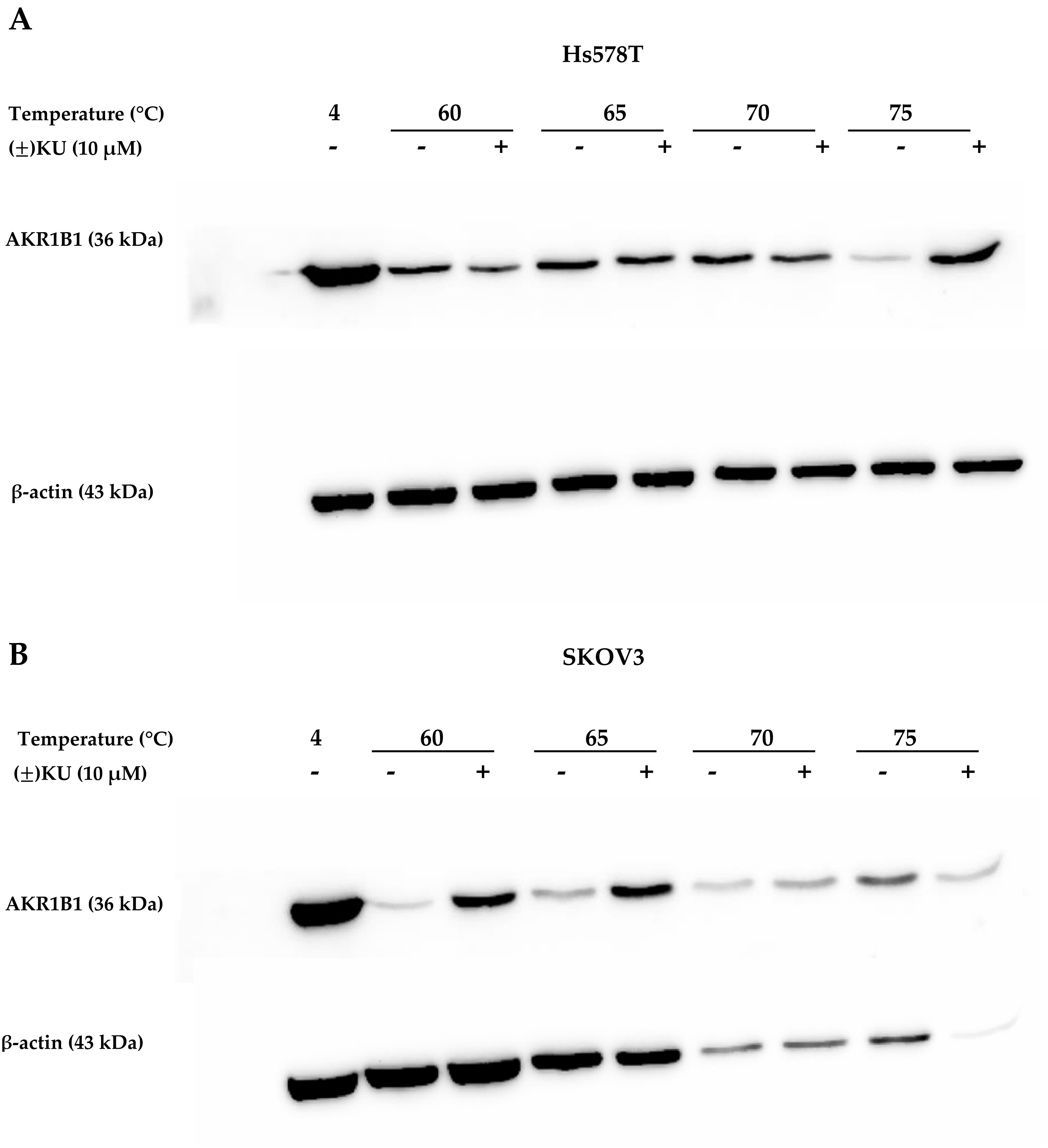

Figure S3. CETSA-based determination of target engagement between (±)KU and AKR1B1 on (**A**) Hs578T cells and (**B**) SKOV3 cells. Both cells were treated with 10 μM of (±)KU at 37 °C for 1 h. Then, cells were harvested and heated at 60, 65, 70 or 75 °C followed by determination of AKR1B1 and β-actin (internal control) using Western blot analysis.

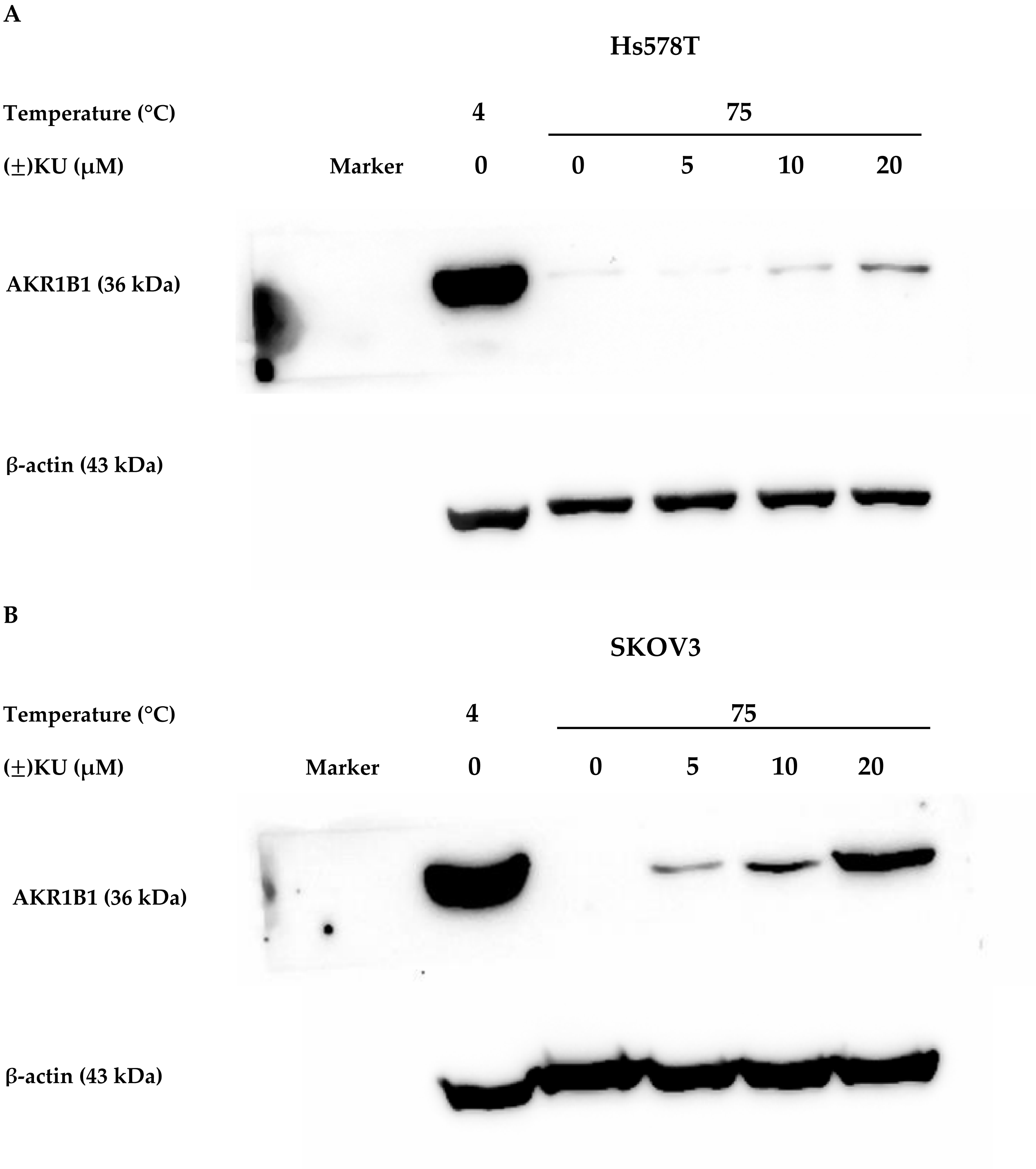

Figure S4. CETSA-based determination of target engagement between (±)KU and AKR1B1 on (**A**) Hs578T cells and (**B**) SKOV3 cells. Both cells were treated with various concentrations of (±)KU at 37 °C for 1 h. Then, cells were harvested and heated at 75 °C followed by determination of AKR1B1 and β-actin (internal control) using Western blot analysis. The relative intensity of protein is represented in Figure 4.

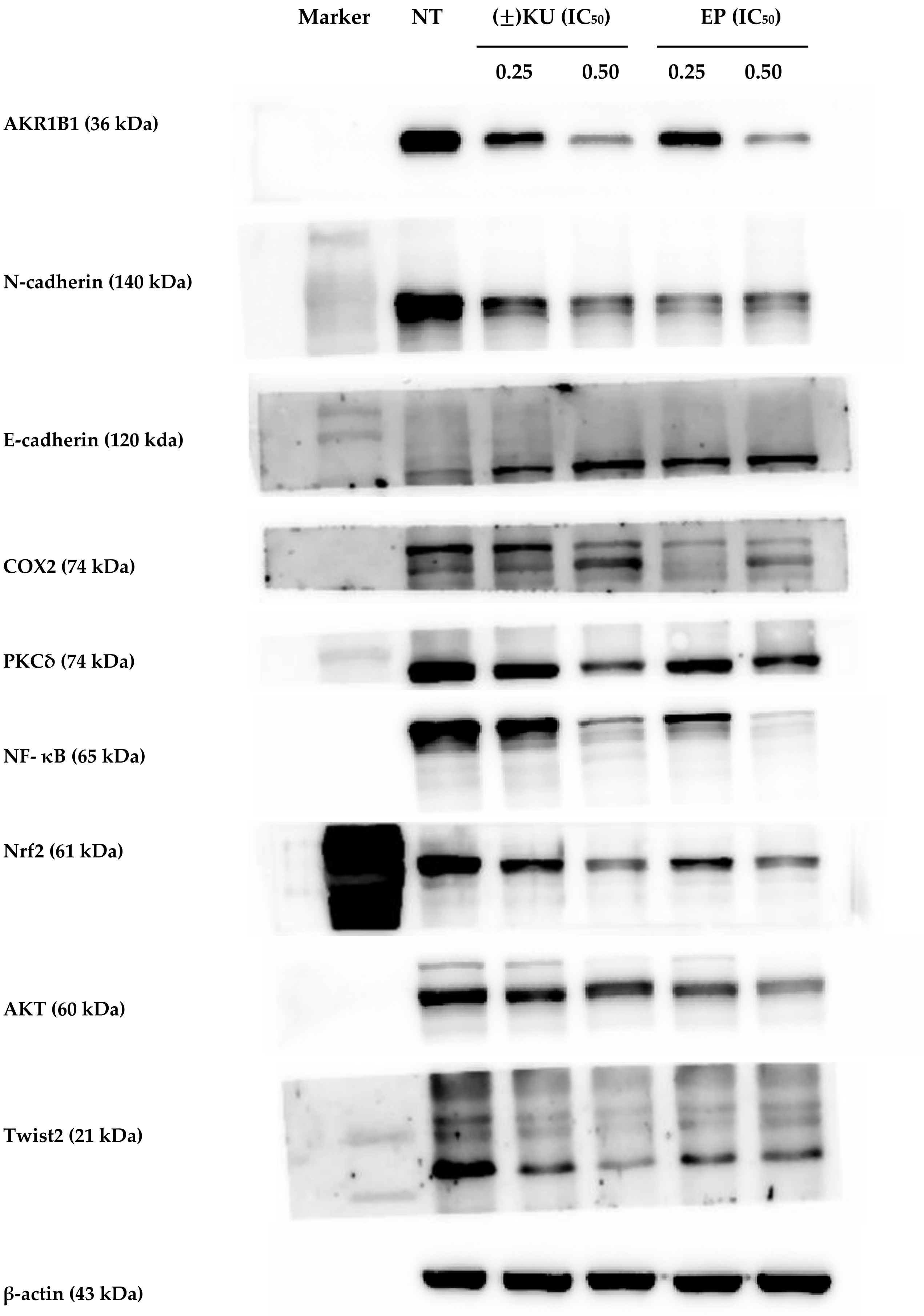
Marker
NT
(±)KU (IC50)
EP (IC50)
0.25
0.50
0.25
0.50
AKR1B1 (36 kDa)
N-cadherin (140 kDa)
E-cadherin (120 kda)
COX2 (74 kDa)
PKCδ (74 kDa)
NF- κB (65 kDa)
Nrf2 (61 kDa)
AKT (60 kDa)
Twist2 (21 kDa)
β-actin (43 kDa)

Figure S5. Effect of (±)KU on AKR1B1 and its downstream signaling molecules. Hs578T cells were treated with (±)KU and EP at a concentration of 0.25× and 0.50× IC_{50} (1.40, 2.79 μM for (±)KU and 24.02, 48.04 μM for EP, respectively) for 48 h. The protein level of AKR1B1 and its downstream molecules (PKCδ, NF-κB, AKT, Nrf2, COX2, Twist2, E-cadherin and N-cadherin) was determined by Western blot analysis. The relative intensity of protein is represented in Figure 6B. NT, non-treated cells; (±)KU, trans-(±)-kusunokinin; EP, epalrestat.

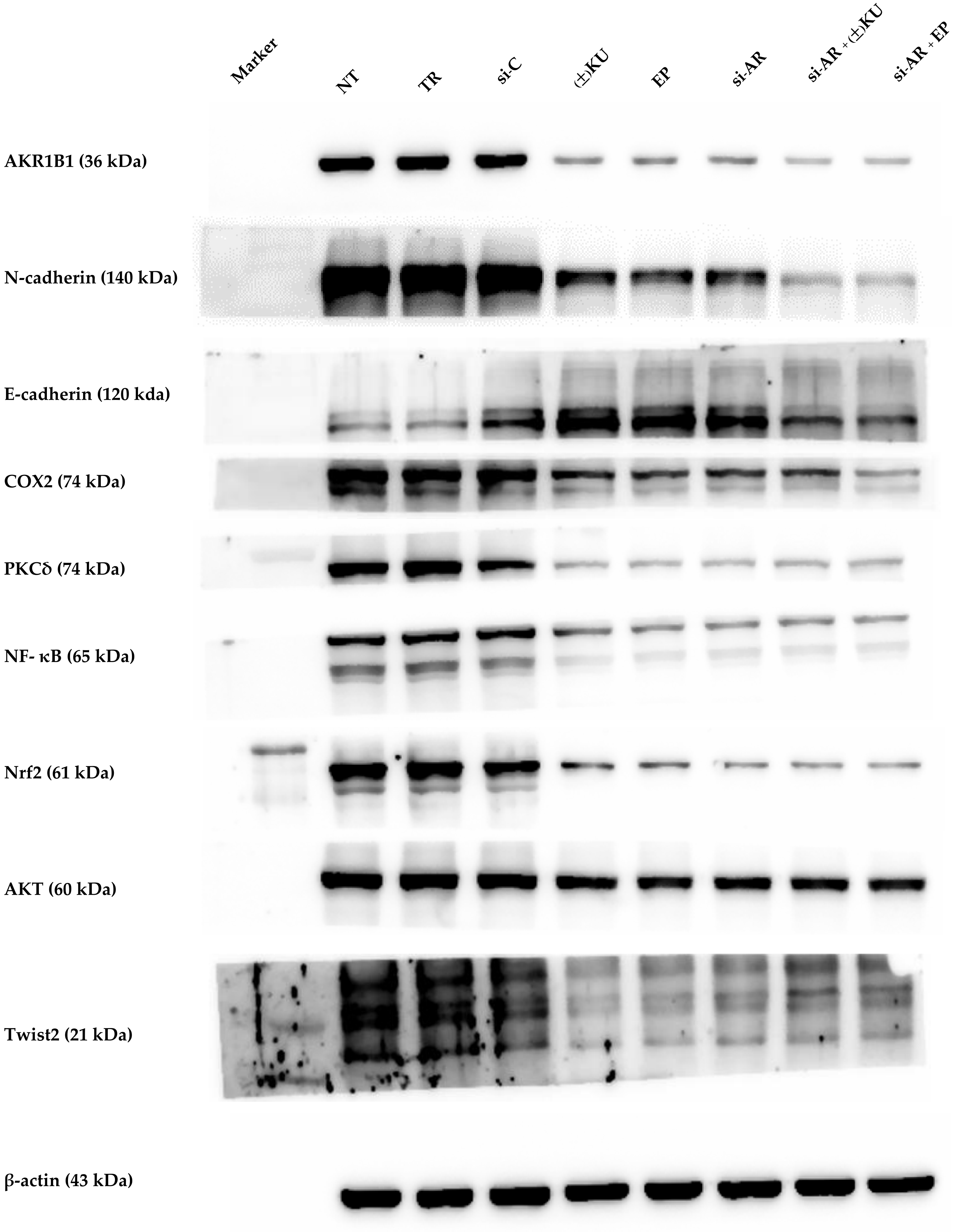
Marker
NT
TR
si-C
(±)KU
EP
si-AR
si-AR + (±)KU
si-AR + EP
AKR1B1 (36 kDa)
N-cadherin (140 kDa)
E-cadherin (120 kda)
COX2 (74 kDa)
PKCδ (74 kDa)
NF- κB (65 kDa)
Nrf2 (61 kDa)
AKT (60 kDa)
Twist2 (21 kDa)
β-actin (43 kDa)

Figure S6. Effect of (±)KU, EP, siRNA-AKR1B1 and combination of compounds with siRNA-AKR1B1 on AKR1B1 and its downstream signaling molecules. Hs578T cells were treated with 2.79 μM (±)KU or 48.04 μM EP or 100 nM siRNA-AKR1B1 or a combination of 100 nM siRNA-AKR1B1 with each compound for 72 h. The protein level of AKR1B1 and its downstream molecules (PKCδ, NF-κB, AKT, Nrf2, COX2, Twist2, E-cadherin and N-cadherin) was determined by Western blot analysis. The relative intensity of protein is represented in Figure 7B. NT, non-treated cells; TR, DharmaFECT 4 transfection reagent; si-C, siGENOME non-targeting control; (±)KU, trans-(±)-kusunokinin; EP, epalrestat; si-AR, siRNA-AKR1B1.
